# An interpretable boosting model to predict side effects of analgesics for osteoarthritis

**DOI:** 10.1186/s12918-018-0624-4

**Published:** 2018-11-22

**Authors:** Liangliang Liu, Ying Yu, Zhihui Fei, Min Li, Fang-Xiang Wu, Hong-Dong Li, Yi Pan, Jianxin Wang

**Affiliations:** 1 School of Information Science and Engineering, Central South University, Changsha, China; 2 Department of Network Center, Pingdingshan University, Pingdingshan, 467000 China; 3 Department of Mechanical Engineering and Division of Biomedical Engineering, University of Saskatchewan, Saskatoon, SK S7N 5A9 Canada; 4 Department of Computer Science,Georgia State University, Atlanta, GA30302 USA

**Keywords:** Osteoarthritis, Analgesics, Cardiovascular disease, XGBoost, Risk features

## Abstract

**Background:**

Osteoarthritis (OA) is the most common disease of arthritis. Analgesics are widely used in the treat of arthritis, which may increase the risk of cardiovascular diseases by 20% to 50% overall.There are few studies on the side effects of OA medication, especially the risk prediction models on side effects of analgesics. In addition, most prediction models do not provide clinically useful interpretable rules to explain the reasoning process behind their predictions. In order to assist OA patients, we use the eXtreme Gradient Boosting (XGBoost) method to balance the accuracy and interpretability of the prediction model.

**Results:**

In this study we used the XGBoost model as a classifier, which is a supervised machine learning method and can predict side effects of analgesics for OA patients and identify high-risk features (RFs) of cardiovascular diseases caused by analgesics. The Electronic Medical Records (EMRs), which were derived from public knee OA studies, were used to train the model. The performance of the XGBoost model is superior to four well-known machine learning algorithms and identifies the risk features from the biomedical literature. In addition the model can provide decision support for using analgesics in OA patients.

**Conclusion:**

Compared with other machine learning methods, we used XGBoost method to predict side effects of analgesics for OA patients from EMRs, and selected the individual informative RFs. The model has good predictability and interpretability, this is valuable for both medical researchers and patients.

## Background

In order to control or prevent the diseases, people try to use disease similarity and drug repositioning to gain deeper insights into pathogenic mechanisms of complex diseases [1–3]. However, in clinical practice, there are particularity and complexity between similar diseases, and there are limitations in drug repositioning. In recent years, the rapid increase of the EMRs provide a new direction for assessing the determinants of the drug used and predict the progress of the disease. The prediction of drug side effects based on EMRs is a crucial task for epidemiology and public health. Most of the time, the side effects of the drug are not immediately apparent. Some drugs may be silent but can cause significant mortality and morbidity after onset. In the process of treatment, appropriate or preventive medication reduces not only the impact of diseases on the quality of life, but also the burden of medical expenses. In this study, we focus on predicting side effects of analgesics for OA patients based on EMRs.

OA is the most common joint disease for middle-aged and elderly people worldwide [4]. In the past few decades, OA has been recognized as a well-defined disease that affects over 75 million people in the United States, Europe and Japan Organization. By 2020, OA is predicted to become the fourth leading cause of disability globally [5]. It causes chronic joint pain and reduces physical functioning, knee OA is the most common subtype. Almost all patients struggle with the long-term pain. Standard treatments usually begin with non-pharmacologic approaches for symptom relief and functional recovery, including weight loss, diet, exercise, physical therapy and orthotic devices [6, 7]. Analgesics are widely used in the treatment of OA. Analgesics and nonsteroidal anti-inflammatory drugs (NSAIDs: one type of analgesics) play a major role in chronic OA pain relief [8]. Those drugs are the most widely used in the treatment of arthritis. While relieving pain, those medicines also increase both systolic and diastolic blood pressure, and can precipitate congestive cardiac failure and myocardial infarction [9–11]. Some patients have an elevated cardiovascular risk without the presence of any symptoms or history. Bally et al. searched Medline, Embase, and PubMed by applying filters for retrieval of observational studies, and they pointed out that the inappropriate use of analgesics led to an increase of 20 to 50% of cardiovascular diseases, even if some patients have no symptoms or previous medical history [11].

Currently, there are neither known cures for OA, nor effective interventions to slow disease progression [12]. Recently, many machine learning and deep learning methods have been applied to OA EMRs data mining, those methods are shown to be superior to the conventional methods in the specific tasks, classification and prediction, such as logistic regression method was used to predict the risk of knee OA [13]; deep convolutional neural networks (CNN) was used to quantify the severity of knee osteoarthritis, and showed a sizable improvement on the current state-of-the-art methods [14]; support vector machine(SVM) was used to predict symptomatic progression of OA using the texture metric [15], etc. However, there are few studies on the side effects of OA medication, especially for the risk prediction model of side effects of analgesics. Therefore, it is of great significance to identify these asymptomatic patients before opening the painkillers to assist in clinical medication. In addition, most prediction models do not provide clinically useful interpretable rules that could explain the reasoning process behind their predictions. They only produce the accuracy score, precision score, recall score that describe the chance of patients fall ill, sometimes those metrics only reflect the one side performance of the prediction models. Especially on imbalanced or skewed data sets, those evaluation metrics do not respond to the true performance of the model.

In this study, based on Gradient Boosting Decision Tree (GBDT) [16] technique we propose a scalable end-to-end tree boosting algorithm called eXtreme Gradient Boosting (XGBoost) [17]. XGBoost is widely used by data scientists to overcome many machine learning challenges. In XGBoost, individual trees are created using multiple cores and data is organized in order to minimize the lookup times. We use this approach to study the side effects of analgesics for Osteoarthritis disease in two aspects: the prediction of side effects of analgesics on cardiovascular disease and risk feature selection. In XGBoost algorithm, the sparsity-aware algorithm is used to handle sparse data, the weighted quantile sketch is used to predict the side effects of analgesics on cardiovascular diseases, and the splitting nodes algorithm is used to get the importance of each splitting node (feature) in a tree. Our method builds on the Osteoarthritis Initiative (OAI) dataset (https://oai.epi-ucsf.org/datarelease/default.asp). We test the samples in the OAI dataset to predict whether a patient is suitable to use analgesics. In the end, we combine the characteristics of the prediction model to identify high-risk features of cardiovascular diseases caused by analgesics, and analyze informative RFs, which can provide decision support for the use of analgesics in OA patients.

## Methods

### Data

The data we used come from OAI which is sponsored by the National Institutes of Health (NIH, part of the Department of Health & Human Services). NIH is a nationwide academic unit, which helps researcher better understand how to prevent and treat knee osteoarthritis. OAI is a public domain research resource to facilitate the scientific evaluation of biomarkers, and for osteoarthritis as potential surrogate endpoints for disease onset and progression. The OAI establishes and maintains a natural history database for osteoarthritis that includes clinical data, X-ray images, MRIs, and a biospecimen repository from 4796 men and women at ages of 45-79 enrolled between February 2004 and May 2006. Datasets are available for public access through the OAI website at the http://www.oai.ucsf.edu. Details of the participants, enrollments, evaluations and follow-ups are also available for public access at the OAI website.

The OAI dataset collect various pieces of information about patients, including demographic features (e.g. age, sex etc.); medical history; physical measurements (e.g. blood pressure etc.); nutrition (e.g. food frequency, vitamin etc.); physical exam, measurements (e.g. physical exam, joint exam etc.); medication inventory (e.g. aspirin, NSAID etc.); X-ray and MRI images, etc.. In our study, we used all the diagnostic data except for the image data.

### Data controls and pre-processing

In this study, we predicted whether OA patients are suitable for using analgesics. At first we confirmed OA patients and combined clinical record data with Kellgren-Lawrence (KL) grades. The KL grading system is considered the gold standard for initial assessment of knee osteoarthritis severity in radiographs [18, 19]. KL grades come from X-ray images examination. The KL grading system has 5 grades to indicate radiographic knee OA severity. “Grade 0-4“ represents normal, doubtful, minimal, moderate, severe and respectively. We checked the “Medication Inventory Form“ (MIF) of each participant who used analgesics and KL grades in 1-4, and filtered out those who have had cardiovascular diseases at the first visits.

Data pre-processing is an important step for the machine learning model as data with good quality can improve the model performance. In our study, several data pre-processing steps were adopted as follows:

1. For all records of the patient, each records interval must be more than 18 months, since the 18 months is more than the 12 months of the analgesic remaining in vivo.

2. Patients with inconsistent and/or error components, such as death, or quit, were excluded.

3. We removed those feature variables with > 50% missing rates, and some missing values were imputed by the hot-deck method [20], other missing values were imputed by the same variable in prior or later observations of the specific patient in the data set. Such as age missing, or weight missing.

4. The KL grades of each participant is in 1-4, and we used the KL grades as the label for OA diagnosis.

In the end, we obtained 4350 out of 4796 participants who took analgesics, and 371 out of 4350 participants had cardiovascular disease after they used analgesics in the process of treatment of OA. In our model, each participant with more than 300 features as input to scatter into 2 categories.

### XGBoost model

In this section, we first briefly described the overview of some prediction models. Then we used predication performance and interpretability as core conditions to select machine learning methods. Finally, we used XGBoost model focusing on the prediction and informative RFs selection for side effects of analgesics on OA diseases.

**Fig. 1:**
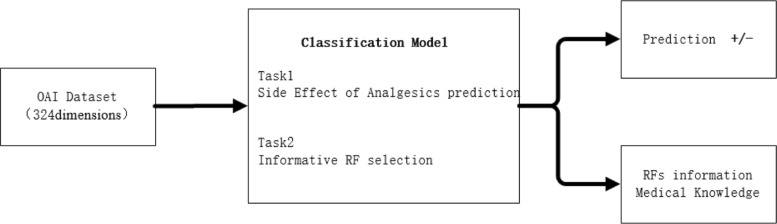
Overview of our framework

All of machine learning and deep learning algorithms can correctly analyze more or less. However, most of methods pay much attention on task prediction or classification, ignoring the interpretability and informative risk factor selection and analysis [21]. Deep learning models need a large number of samples to be trained, and most deep learning algorithms are difficult to explain the process and result to the medical workers. In our study, interpretability is considered as a core requirement for selecting machine learning models in medicine [22]. We define our problems by showing a pipeline for the whole framework. In brief, our proposed system contains two-task functions, as shown in Fig. 1.

The upper component of Fig. 1 shows the roadmap for the first task: side effects of analgesics on the OA disease. The bottom component of Fig. 1 shows the roadmap for the second task: informative RFs selection. Given patients’ information, our method can not only predict the risk of side effects of analgesics on osteoporosis, but also rank the informative RFs and explain the semantics of each RF.

Support Vector Machine (SVM), Logistic Regression (LR), Decision Tree (DT) etc. are classic supervised machine learning models. With SVM, the features are mapped through a kernel function from the original space into a higher-dimensional space. However, this makes the features in the new space not interpretable. In this study, each sample has more than 300 features, and there is no direct relationship between prediction of side effects and the reasons that led to it, which are challenging to LR method. Compared with ensemble learning methods, the decision tree is a weak prediction method, it has the equivalent accuracy to other classic machine learning methods while maintaining interpretability. Boosting method is a popular and effective ensemble learning algorithm in data mining field. By weighting each weak classifier into strong classifier, it can effectively reduce errors and achieve accurate classification results. Gradient Boosting method is based on Boosting, the idea of this method is to continuously reduce residuals and further reduce the residual of previous models in the gradient direction, and get a new model [16]. XGBoost is one type of regularization form of Gradient Tree Boosting besides Regularized Greedy Forest (RGF) [17], which has been shown to give state-of-the-art results on lots of machine learning problems [23, 24]. Regularization is used to control the complexity of the tree to get simpler model and avoid over-fitting in this algorithm. XGBoost also calculates training loss to measure how predictive the model is with using new function additively to the previous prediction, and the results of the algorithm are given by the sum of many tree classifiers. This algorithm could be used for classification, regression, and feature ranking. In this study, we use XGBoost as a model to make a trade-off between predictive power and interpretability.

The main idea of XGBoost method is that the results of the algorithm are given by the sum of many tree classifiers. For a training data set with n samples, the prediction is given by a function of the sum of *K* classifiers

1 $$ \widehat{y_{i}}=F(x_{i})=\sum\limits_{k=1}^{K}f_{k}(x_{i}),f_{k}\in\varphi,(1)  $$

where *x*_*i*_ represents the *i*−*th* sample in the training set, *φ*={*f*(*x*)=*w*_*s*_(*x*)}(*s*:ℜ^*m*^→*T*,*w*∈ℜ^*T*^) is a collection of decision trees, each tree *f*(*x*) corresponds to a structure parameter *s* and leaf weights *w*, *w*_*i*_ is used to represent score on the *i*−*th* leaf. *m* is the number of features, *T* is the number of leaves in the tree. *K* is the number of trees which are used to classify the data set (eg., in our study *K*=2). $\widehat {Y}_{i}$ is the prediction.

XGBoost is a scalable machine learning system for tree boosting. To obtain the minimum loss function, Chen et al. used algorithm to greedily find the penalty which can reduce the most loss. The loss function can be rewritten as

2 $$ {}L^{(t)}\,=\,argmin\!\sum\limits_{i=1}^{n}\! \left[\!l{(y_{i},\widehat{y}^{(t-1)}\,+\,g_{i}f_{t}{(x_{i})})\,+\,\frac{1}{2}h_{i}f_{t}^{2}{(x_{i})\!}}\right]\,+\,\Omega{(f_{t})},  $$

and

$$\begin{aligned} g_{i}&=\partial_{\widehat{y}^{(t-1)}}l{(y_{i},\widehat{y}^{(t-1)})},h_{i}\\ &=\partial_{\widehat{y}^{(t-1)}}^{2}l{(y_{i},\widehat{y}^{(t-1)})},\Omega{(f_{t})}=\gamma{T}+\frac{1}{2}\lambda||{w}||^{2} \end{aligned} $$

where *l*() represents the loss function. *g*_*i*_ and *h*_*i*_ are the first and second order gradient statistics on the loss function, respectively. The last term *Ω*(*f*_*t*_) is the penalty. *γ* and *λ* are two parameters to control the complexity of the tree. The additional regularization term helps smooth the final learnt weights to avoid over-fitting. Intuitively, the regularized objective tends to select a model employing simple and predictive functions.

Chen et al. used Eq. (3) to evaluate the split candidates. The loss reduction after the split is given by

3 $$ {}L_{split}\,=\,\frac{1}{2}\left[ \frac{\left({\sum}_{i\in{I_{L}}}g_{i}\right)^{2}}{\!{\sum}_{i\in{I_{L}}}h_{i}+\lambda}\,+\, \frac{\left({\sum}_{i\in{I_{R}}}g_{i}\right)^{2}}{{\sum}_{i\in{I_{R}}}h_{i}+\lambda}\,-\, \frac{\left({\sum}_{i\in{I}}g_{i}\right)^{2}}{{\sum}_{i\in{I}}h_{i}+\lambda} \!\right]\,-\,\gamma,  $$

where *I*_*L*_ and *I*_*R*_ are the instance sets of left and right nodes after the split, *g*_*i*_ and *h*_*i*_ are the first and second order gradient statistics on the loss function.

In order to get the importance of each splitting node (feature) in a tree *T*, we look for an explanation of how node relative variable importance is computed in XGBoost method. The measures are based on the number of times that a node is selected for splitting, weighted by the squared improvement to the model as a result of each split, and averaged over all trees, the importance of each splitting node defined as

4 $$ \widehat{I}_{j}^{2}{(T)}={\sum}_{t=1}^{J-1}\widehat{i}_{t}^{2}{1(v_{t}=j)},  $$

where the summation is over the non-terminal nodes *t* of the *J*-terminal node of tree *T*, 1() represents the indicator function which is associated with squared-influence. *v*_*t*_ is the splitting variable associated with node *t*, and $\widehat {i}_{t}^{2}$ is the corresponding empirical improvement in squared error as a result of the split, $\widehat {i}_{t}^{2}$ is defined as

5 $$ \widehat{i}_{t}^{2}=i^{2}{(R_{l},R_{r})}=\frac{w_{l}w_{r}}{w_{l}+w_{r}}{(\overline{y_{l}}+\overline{y_{r}})^{2}},  $$

where the mean weight of the left and right children nodes of *t* are expressed $\overline {y_{l}}$, $\overline {y_{r}}$, and *w*_*l*_, *w*_*r*_ are the corresponding sums of the weights. For a collection of decision trees $\{T_{m}\}_{1}^{M}$, obtained through boosting, Eq. (5) can be generalized by its average over all of the trees in the sequence. Equation (5) is redefined as

6 $$ \widehat{I}_{j}^{2}=\frac{1}{M}\sum\limits_{m-1}^{M}{\widehat{I}_{j}^{2}{(T_{m})}}.  $$

### Evaluation metric

In binary classification, accuracy, precision, recall and error rate are commonly used as metrics to measure how well a binary classification correctly identifies or excludes a condition. Accuracy is the proximity of measurement results to the true value; precision (also called positive predictive value) is the fraction of relevant instances among the retrieved instances; recall (also known as sensitivity) is the fraction of relevant instances that have been retrieved over the total amount of relevant instances; error rate is commonly used as the evaluation metric of the classification method performance. Nevertheless, for most skewed medical data sets, above four metrics are not to correctly reflect the prediction results in all the times as the method usually would be misclassifying entire minority samples to the class of majority. In our study, we only use precision, recall and error rate as base metrics, which can be calculated as follows

7 $$ Precision=\frac{TP}{TP+FP},  $$

8 $$ Recall=\frac{TP}{TP+FN},  $$

9 $$ Error Rate=\frac{FP+FN}{TP+TN+FP+FN},  $$

where *TP*, *FP*, *TN* and *FN* denote true positives, false positives, true negatives and false negatives, respectively.

In addition, there are several techniques to assess the performance of binary classification across a range of thresholds on imbalance medical data sets. Two common metrics to evaluate the performance of algorithms are Receiver Operator Characteristic (ROC) curves [25] and Precision-Recall (PR) curves [26]. ROC curve and PR curve are classical evaluation tool for binary classification that allows the visualization of performance at various thresholds. ROC curves are plotted to generally capture how the number of correctly classified abnormal cases varies with the number of incorrectly classified normal cases as abnormal cases. In addition to the ROC curve, we use the PR curve as another important metric. PR curves are also plotted to respond the fraction of examples classified as abnormal cases that are truly abnormal, PR curves are visual representations of the performance of a model in terms of the precision and recall. On the imbalanced or skewed data sets, PR curves are a useful alternative to ROC curves that can highlight performance differences that are lost in ROC curves [27]. In case of binary classification, ROC curve and PR curve can be compared quantitatively by the area under curve (AUC). It is noteworthy that AUC is not sensitive to whether the sample categories is balanced. The AUC indicates the performance of a classifier: The larger the value of AUC is, the better a classifier performs.(an AUC of 1.0 indicates a perfect performance).

## Results and discussion

### Comparison to other classification methods

In this section, we show the RFs prediction using records in OAI dataset. To investigate the efficacy of XGBoost method, we choose classic supervised machine learning models C4.5 decision tree (DT), SVM, LR and GBDT as comparison models. The detailed results of 10-fold cross-validation are reported in Table 1. We can see that DT model gets the highest precision score, compared with the other four methods, there is no advantage in recall score and error rate score for DT model. In the 5 models, although XGBoost and GBDT models are not the best on the precision scores, the two models are the most balanced performance in the three metrics compared with three other models. The error rates of XGBoost and GBDT are equal, the precision score of GBDT is higher than that of XGBoost, and the recall score of XGBoost is higher than that of GBDT. In our study, precision score and recall score are main measures for the performance of the models. Typically, precision and recall are inversely related, i.e. as precision increases, recall decreases and vice-versa. A balance between these two metrics needs to be achieved by the IR system, and to achieve this and to compare performance, the addition metrics come in hand. In order to compare the predication performance of XGBoost and GBDT, physicians more concern whether they can correctly identify the real patients, we use AUC-ROC and AUC-PR as the additional metrics.

**Table 1:** Performance comparison among the 5 different classifiers: Linear Regression (LR), Supported Vector Machine (SVM), Decision Tree (DT), Gradient Boosting Decision Tree (GBDT), eXtreme Gradient Boosting (XGBoost)

	LR	SVM	DT	GBDT	XGBoost
Precision	0.75	0.64	0.97	0.89	0.81
Recall	0.69	0.75	0.5	0.75	0.84
Error rate	0.04	0.04	0.08	0.03	0.03

**Fig. 2:**
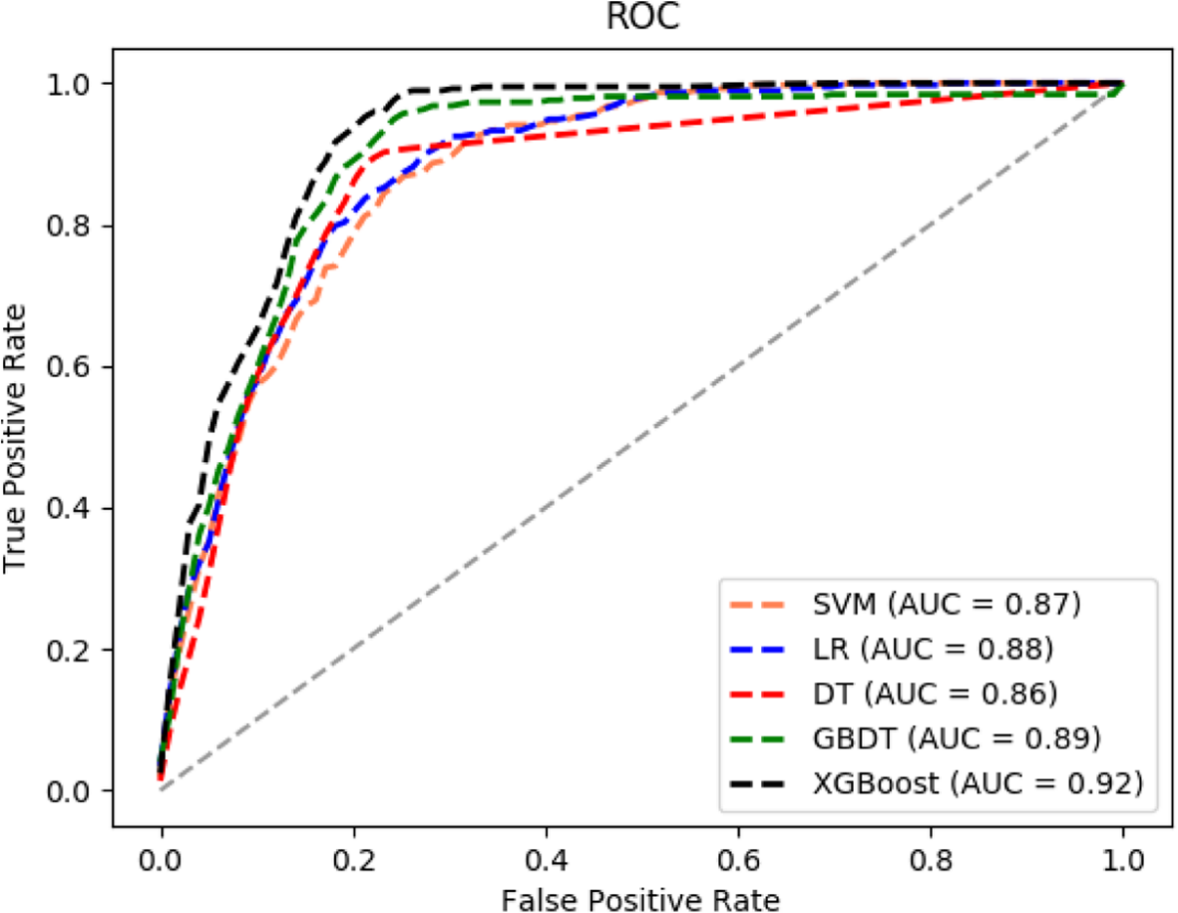
ROC curves of 5 models

We integrate all features of OA patients in the EMRs since they contain the characteristics of data itself and can be nicely tuned with under the help of two kinds of labels. We compare the ROC curves and PR curves of 5 models. All ROC curves are shown in Fig. 2. As illustrated by a legend in Fig. 2, the lines of each color in the illustration correspond to different models. Compared with the other four curves, AUC value of the black curve is little larger than the other four curves, it means that the XGBoost model obtains the best performance in 5 models. Although the performance of XGBoost is the best one among 5 models, it can be seen that the 5 curves are very close.

**Fig. 3:**
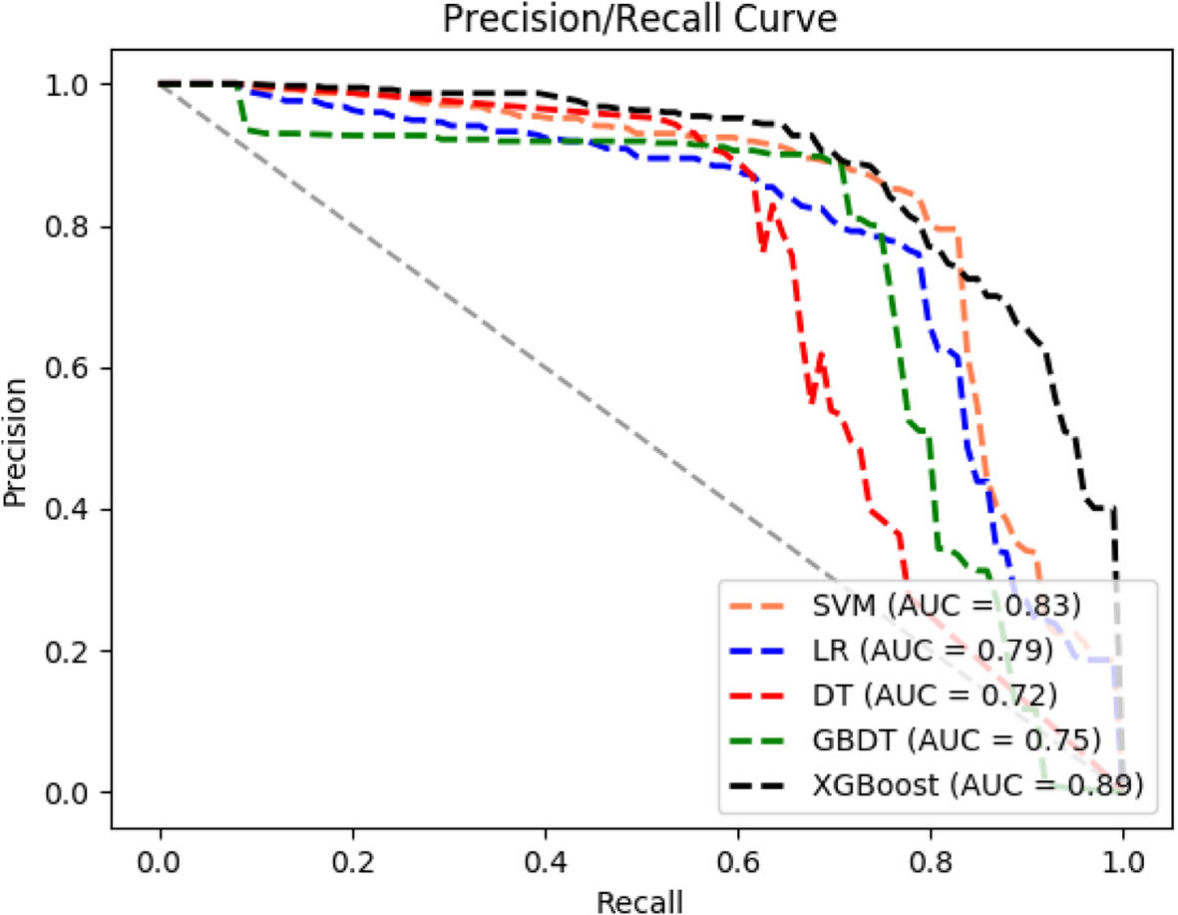
PR curves of 5 models

In order to further compare the performance of the 5 models, we use PR curve as another metric. The PR curve for each predication model is shown in Fig. 3. The PR curves are increasingly used in the machine learning community, particularly for imbalanced data sets where one class is observed more frequently than the other class. The curve plots the precision (positive predictive value) against the recall (true positive rate) and is equivalent to the false discovery rate curve. The PR curve value is the better method performs as shown is Fig. 3, AUC of the black curve is much obviously larger than the other curves. With reference to a legend, the black curve represents the XGBoost model, it means XGBoost model gets the best result among the 5 models.

In summary, compared with the other four classic machine learning models, the precision, recall, error rate of XGBoost model are well balanced, and XGBoost model achieved the best results on both the ROC curves and PR curves evaluation metrics. It means that on the skewed OAI dataset, the XGBoost method is better than the other four models in terms of AUC-ROC and AUC-PR.

### Informative RFs and interpretability

Aside from the above metrics, interpretability is another vital component in computer aided diagnosis (CAD). First, a good prediction model should clearly show the decision processes to medical workers, and suggest that feasible improvements [28]. Second, the model with sound interpretability can overcome the reluctance of replacing traditional statistical methods, such as visualization graphs and result tables. However, interpretability tends to be ignored in many studies for two reasons. First, popular base models (e.g., SVM, LR) are inherent black-box systems. Second, ensemble methods, such as bagging, boosting and stacking, also address problems in establishing prediction models.

As tree-boosting models, the XGBoost-based side effect prediction models are composed of classification trees as base models. The inherent interpretability of weak decision trees reduces the complexity of the models, thus enhancing the interpretability of the entire model. The interpretability of the XGBoost-based model mainly lies on selecting feature importance.

**Fig. 4:**
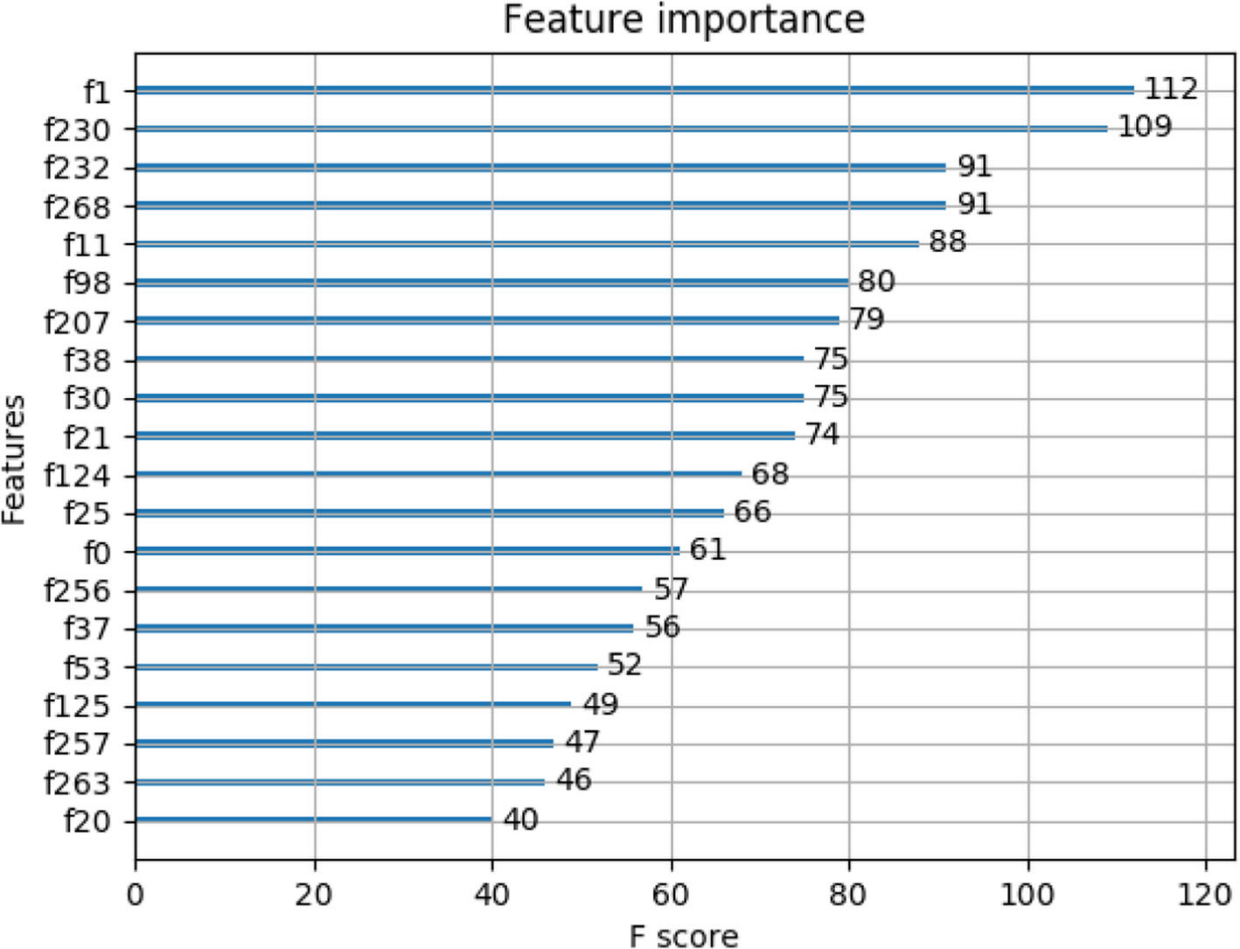
Feature importance plot of risk features

In the previous section, we have used XGBoost model to predict side effects of analgesics for OA patients. In this section, we aim to capture the informative RFs of OA patients in OAI. As show in Fig. 4 our method selected 20 RFs by splitting nodes algorithm which sums up the number of each important splitting node (feature) in trees. The higher F score implies that the corresponding feature is more important. We seek to identify experimental results in literature from the biomedical database. Each description of feature can be found from the data provider website(https://oai.epi-ucsf.org/datarelease/default.asp). The side effect of analgesics appears to be associated with several known RFs that are well described in the literature.

As shown in Table 2, feature descriptions come from the OAI dataset provider, according to the OAI documents, the 20 RFS are divided into 5 categories: Demographics, Anthropometry, Comorbidity, Blood Measures and Physical activity Measures. In Demographics and Anthropometry categories, f1, f11, f98, f124, f125, f268 indicate age, bmi, height, mental health grade, physical health grade, weight, respectively; In Comorbidity category, f30, f53, f232 indicate comorbidity; In Blood Measures category, f21, f25, f230 indicate the different value of blood pressure; In Physical activity Measures category, f0, f20, f37, f38, f207, f256, f257 and f263 indicate the different values of physical activity or performance measures. The RFs in Demographics and Anthropometry categories are commonly used parameters to measure the condition of a patient, these 6 RFs are prevalence of factors contributing to the broader clinical problem of pain and disability [29] and have already been used to evaluate the risk of patients in most diseases. In this study we much more concerned other 3 categories:

**Table 2:** The description of 20 RFs from OAI dataset documents

Features	Name	Category
f1	Age	Demographics
f230	Radial pulse	Blood Measures
f232	Asthma	Comorbidity
f268	Weight	Anthropometry
f11	Body mass index(bmi)	Anthropometry
f98	Height	Anthropometry
f207	Physical activity scale	Physical activity Measures
f38	Repeated chair stands time1	Physical activity Measures
f30	Operation to unclog or bypass arteries in legs	Comorbidity
f21	Diastolic	Blood Measures
f124	Mental summary scale	Anthropometry
f25	Systolic	Blood Measures
f0	Walk pace	Physical activity Measures
f256	20-meter walk steps1	Physical activity Measures
f37	Repeated chair stands time2	Physical activity Measures
f53	Diabetes	Comorbidity
f125	Physical summary scale	Anthropometry
f257	20-meter walk steps2	Physical activity Measures
f263	20-meter walk time	Physical activity Measures
f20	Heavy housework	Physical activity Measures

#### 1) Analgesics and comorbidity

As shown in Table 2, the feature f30 indicates operation to unclog or bypass arteries in legs, a large number of analgesics are needed after the operation. The features f53, f232 indicate diabetes, asthma, respectively. As report in EMR, most of 371 cardiovascular disease participants have one or more comorbidities. Those comorbidities appear to be a metric for analgesics used, some study have been well described in medical literature. In the study of Kerkhof et al., OA care institutions and medical researchers believe that analgesics and other diseases (such as asthma, diabetes, cancer, renal) are also closely related [30]. In 2013, Bhala et al. published their research results in The Lancet: Coxibs (one of analgesics) and NSAIDs are associated with an increased risk of cardiovascular disease and upper gastrointestinal complications [31]. The Scientific Advisory Committee of the National Kidney Foundation recommended analgesics not as the drug choice for patients with impaired renal function [32]. In our experiment, as shown in Table 2, asthma, diabetes are closely linked with cardiovascular disease, In 2000, Landewe et al. reported these diseases had a detrimental effect on cardiovascular disease [33]. Physicians should be more careful in using analgesics for patients with these comorbidities.

#### 2) Analgesics and blood measures

In Table 2, in addition to those comorbidity informative risk features, our model also identifies some RFs related to blood pressure, (e.g., f21, f25, f230), including diastolic, systolic and radial pulse, which are important risk factors for cardiovascular disease. It is well known that blood pressure is closely related to cardiovascular disease, blood pressure is a secondary risk factor in that blood pressure pills may increase the risk [9, 10], the analgesics can have a direct side effect on blood pressure. For patients with abnormal blood pressure, they should be careful in using analgesics.

#### 3) Analgesics and physical activity measures

In the study of Bijlsma et al., specific exercises can reduce pain and improve function in patients with OA of the lower limbs [8]. However, the f0, f20, f37, f38, f207,f256, f257, f263 indicate walking pace, standing time, walking distance, heavy housework etc., all those are closely related to life habits, and not specific exercises designated by physician. Lifestyle-related behavioral and environmental risk factors are also important causes of analgesics drugs used [8]. The knee functions and physical activities (e.g. standing, walking, housework) are easy to obtain, and can be easily incorporated into routine clinical practices.

Based on the features shown in Table 2, these factors need to be taken into consideration when a patient is given an analgesic for OA. In this study, we only select the top 20 informative RFs to analyze. Some discarded RFs might still make contribution to enhancing the predictive behaviors.

## Conclusions

We studied the prediction of side effects of analgesics on cardiovascular diseases in the OA treatment. The complex and highly relevant relationship between the risk factors are inevitable, it is a challenge to our method. In this study, based on the OA EMRs, we used XGBoost method to predict patients’ use of analgesics, and selected the individual informative RFs. This is valuable for both medical researchers and patients. We selected top 20 informative RFs and analyzed the relationship among the RFs, analgesics and cardiovascular disease from the medical literature. In future, we would combine the EMRs with the MRI images to analyze the OA disease. We would also try to predict drug indications by integrating related data sources and validated information of drugs and diseases using matrix completion method [34, 35].
